# Factors Affecting Awareness About E-Health Services in Saudi Arabia

**DOI:** 10.7759/cureus.37011

**Published:** 2023-04-01

**Authors:** Haya AlSalloum, Haya M Almalaq, Mohammad J Alyamani

**Affiliations:** 1 Clinical Pharmacy, King Saud University, Riyadh, SAU; 2 Pharmacy, King Saud University, Riyadh, SAU; 3 Pharmacy, AlMaarefa University, Riyadh, SAU

**Keywords:** awareness, satisfaction, 937, ehealth, telemedicine

## Abstract

Aim

Telemedicine or using e-health applications was maximized during the COVID-19 pandemic. This study aimed to explore awareness and satisfaction with several e-health services provided by the Ministry of Health (MOH), including Seha, Moed, 937 Services, and Wasfati.

Methods

A population-based social media survey assessed awareness and satisfaction with these applications. The survey gathered information on demographic and socioeconomic characteristics. Binary logistic regression was used to highlight factors that affect awareness of and satisfaction with these services and thus could be a target for future development.

Results

Overall, 1333 surveys were completed; most (70%) of the participants were female, 44% were between the ages of 18 and 24 years, 83% were of Saudi nationality, and 70% had university degrees or above. Awareness was greatest with the 937 Services, Seha, Moed, and Wasfati applications. Satisfaction was the highest with the Moed application. Factors affecting awareness and satisfaction included age, sex, nationality, and education.

Conclusion

Awareness of and satisfaction with the four major e-health applications were high. This indicates the readiness of the Saudi population to embrace advances in telemedicine in alliance with the Saudi 2030 vision.

## Introduction

Although the traditional method for delivering healthcare is important, telehealth is an alternative with benefits that are maximized in special situations, such as during the 2019 COVID pandemic [1,2]. Cassar et al. announced that the Community COVID-19 Initial Assessment Team (CCIAT) project achieved its goals of safe risk stratification of patients with COVID-19, safe management on an outpatient basis, and appropriate and efficient use of hospital resources [3]. In addition, the idea of telehealth was explored from the patient's perspective. Patients have reported that the benefits of virtual assessment included reducing the waiting time and less delay in treatment and diagnostic workup. They also highlighted reduced anxiety due to delays and cancellations of appointments. Although the study was conducted four years before the COVID-19 pandemic, they reported high patient satisfaction with telehealth compared to face-to-face appointments [4].

The use of telemedicine during the COVID-19 pandemic has been supported by international bodies, such as the World Health Organization (WHO) ​[5]. Telemedicine is a rapidly evolving field that requires flexibility and creativity in response to challenges.

In alignment with the Saudi Vision 2030, Saudi Arabia aims to deliver better health-related services to its people and provide the health department with the budget required to improve health issues [6].​ Government-lead institutions have implemented the e-health system throughout the country to promote better and more accessible health services. The COVID-19 pandemic has changed the world, including the Kingdom of Saudi Arabia (KSA), has stretched various healthcare systems to their limits, and has accelerated the advancement of telemedicine.

Although telehealth was implemented, some obstacles were faced, and these were similar to those encountered in other parts of the world. The challenges were mainly related to the lack of a relationship between doctors and patients, concerns regarding the privacy of patients' health data, medical customs, behaviors of KSA citizens, and no clearly administered regulations ​[7].​ Alanazy et al.. reported a high level of acceptance and satisfaction with the Ministry of Health (MOH) application (Mawed), indicating community readiness to adapt and utilize such services [8]. Although the public's attitude seems to be positive towards e-health use, some reluctance still exists and needs to be addressed with further promotion and education ​[8].

There was a definite change in the attitudes of healthcare consumers toward e-health, and changes were mainly influenced by the pandemic and slightly influenced by self-interest in adoption [9]. To increase the usability of e-health platforms, several factors must be emphasized to increase the number of end users, that is, the patient (consumer), including trust and provided information quality, ease of use, and applicability [10]. Therefore, this study aims to examine how satisfied patients in Saudi Arabia are with e-health services and the platforms available to them. This study also aimed to evaluate consumers' and patients' satisfaction with MOH e-services, including Moed (or Mawed), Seha, 937 Services, and Wasfati.

## Materials and methods

Study design

This was a cross-sectional population-based survey that collected data using a predesigned form. The study is observational in nature and is guided by the strengthening of the observational studies in epidemiology (STROBE) checklist. The data collection process was conducted through a survey distributed via email and different social media platforms [11]. 

Setting

An email was sent through the Almaarefa University administration to all enrolled participants. Additionally, the research team posted a survey link through private social media accounts (Instagram, Twitter, and Snapchat). Participants were then asked to send it to other contacts on social media in a process known as snowball recruitment ​[8]. 

Participants and recruitment

The study targeted adult participants (>18 years), including Saudi and non-Saudi nationals, who were able to answer the survey questions. The survey link was sent via university email and posted on the research team’s private social media accounts. The survey link was opened for recruitment between February and April 2021. 

Variables, data source, and measurements

The survey was predesigned using Google Forms (Google, Mountain View, California), subjected to phase validity by two experts, and piloted on 10 students prior to distribution. The survey had several parts, including a consent form, followed by general questions on demographic characteristics. Other survey items included questions on awareness of different MOH electronic platforms: 937 Services, Wasfati, Seha, and Moed. This was followed by a four-item Likert scale for satisfaction with these services. In Saudi Arabia, 937 Services is a telephone service that was launched in 2018 that provides multiple care services to people who call, including consultation, diagnosis, and prescription ​[12]. Seha is a mobile application that gives people access to medical services, such as sick leave and requests for medications that are unavailable in the country​ [13]. Moed (or Mawed) is a mobile application used to book appointments in government-supported primary care and has been changed to the Sehhati app​ [14,15]. ​ Finally, the Wasfat website enables consumers to receive prescriptions from their nearest accredited community pharmacy [​16].

Bias

Data recruitment included a valid method of recruitment (snowballing), and the analysis was performed using a complete case. 

Study size

Using a proportion of 47% reported by a study done by Alharbi et al. on the use of the Seha application in Saudi Arabia, the calculated population size was 383, with a 95% confidence level and a 5% margin of error. 

Statistical analysis

The survey items were exported from Google Forms to Statistical Package for Social Sciences (SPSS) version 28.0 (IBM Inc., Armonk, New York) [16]. Data were categorized and coded whenever necessary. Crosscheck tabulation was performed to confirm data accuracy. Data are reported as numbers and percentages for categorical variables and mean standard deviation for continuous variables. The unadjusted odds ratio (OR) was used to determine factors affecting awareness. A p-value of <0.05 was considered significant. 

Ethical consideration

This study was approved by the institutional review board of Almaarefa University (approval number: 06-02032021). The participants provided electronic consent once they opened the survey. No participant identifiers were collected; thus, the data were completely anonymous. 

## Results

**Figure 1:**
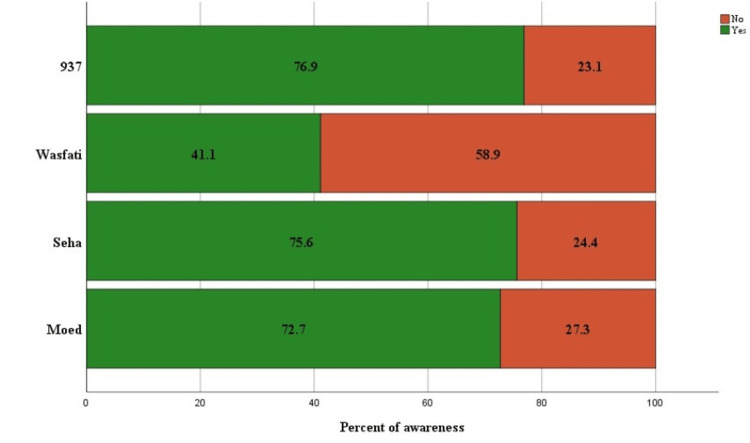
Awareness with the Ministry of Health e-health services, with green indicating awareness

**Figure 2:**
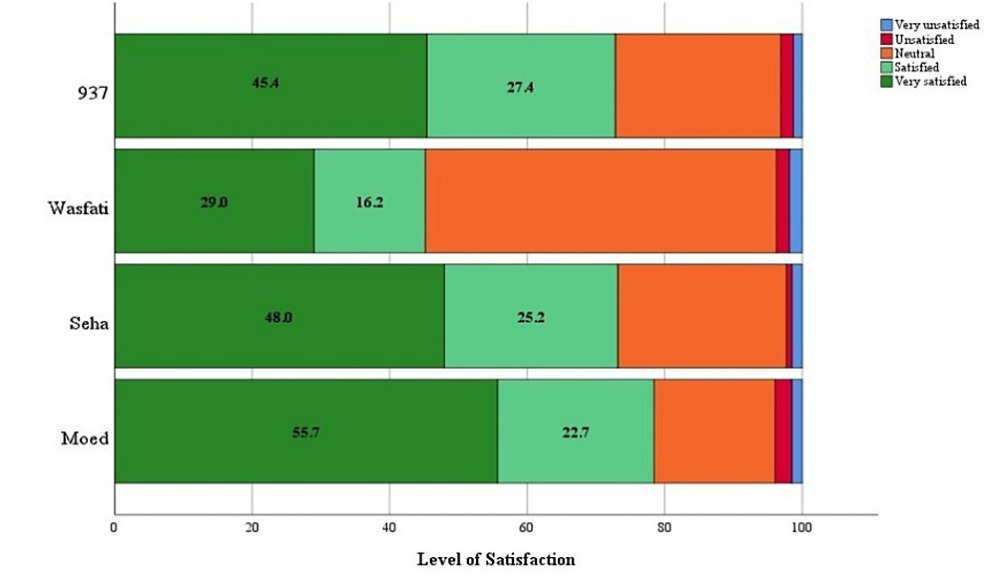
Level of satisfaction of participants with different e-health services, with green indicating high satisfaction

A total of 1333 participants completed the survey. The majority were female (70%), aged 18-24 years of age (44%), of Saudi nationality (83%), and single (61%). With regard to education, most participants had a university degree or higher than 70%. The highest percentage of employees is 37%. Demographic data are presented in Table 1. When evaluating the level of awareness of different MOH e-services, the highest awareness was in 937 Services; 77% of participants were aware of such services. This was followed by Seha at 76%, Moed (or Mawed) at 73%, and Wasfati at 41%. The awareness levels are shown in Figure 1. Participants were mostly satisfied with different services, where the highest level of satisfaction was with Moed (or Mawed) (78%); this was followed by Seha (73%), 937 Services (73%), and Wasfati (45%) (Figure 2). 

**Table 1:** Demographic characteristics of the study population

N=1333		N	%
Gender	Female	931	69.8
	Male	402	30.2
Age (years)	18–24	589	44.2
	25-35	385	28.9
	36-45	192	14.4
	>45	167	12.5
Nationality	Non-Saudi	231	17.3
	Saudi	1102	82.7
Marital status	Single	813	61.0
	Married	520	39.0
Children	No	864	64.8
	Yes	469	35.2
Education level	Primary/intermediate school	45	3.4
	High school	346	26.0
	University and above	931	69.8
	Illiterate	11	0.8
Employment	Student	489	36.7
	Public sector employee	247	18.5
	Privet sector employee	234	17.6
	Retired	60	4.5
	Housewife	126	9.5
	Unemployed	145	10.9
	Self-employed	32	2.4

We explored factors affecting awareness using binary logistic regression and observed that 937 Services was affected by age, with most of the awareness being among people >45 years of age (OR, 2.4; 95% CI, 1.5-3.8; p<0.001), followed by 36-45 years (OR, 2.3; 95% CI, 1.5-3.6, p<0.001). Other factors include Saudi nationality (OR, 1.4; 95% CI, 1.0-2.0; p=0.031), being married (OR, 1.7; (95% CI, 1.3-2.2; p<0.001) and having children (OR, 1.9; (95% CI, 1.42.5; p<0.001). Education seemed to be inversely related to awareness of 937 Services, with those who had a university level of education having an awareness of 0.15 (95% CI, 0.04-0.64; p=0.01). However, factors including being a student, retired, or unemployed increased the likelihood of being aware of 937 Services (Table 2).

**Table 2:** Factors influencing awareness of the Ministry of Health e-health applications 937 Services and Wasfati * indicates a significance based on a significance level of <0.050

N=1333		937 Services			Wasfati		
		Yes (%)	Odds ratio (95% CI)	p-value	Yes (%)	Odds ratio (95% CI)	p-value
Gender	Female	702 (75.4)	Reference		344 (36.9)	Reference	
	Male	323 (80.3)	1.334 (1.000-1.778)	0.050	204 (50.7)	1.758 (1.388-2.227)	<0.001*
Age	18-24	422 (71.6)	Reference		175 (29.7)	Reference	
	25-35	296 (76.9)	1.316 (0.978-1.771)	0.070	168 (43.6)	1.832 (1.401-2.394)	<0.001*
	36-45	164 (85.4)	2.318 (1.494-3.595)	<0.001*	100 (52.1)	2.571 (1.842-3.590)	<0.001*
	>45	143 (85.6)	2.358 (1.477-3.765)	<0.001*	105 (62.9)	4.006 (2.795-5.744)	<0.001*
Nationality	Non-Saudi	165 (71.4)	Reference		72 (31.2)	Reference	
	Saudi	860 (78.0)	1.421 (1.033-1.956)	0.031*	476 (43.2)	1.679 (1.240-2.273)	<0.001*
Marital status	Single	597 (73.4)	Reference		289 (35.5)	Reference	
	Married	428 (82.3)	1.683 (1.280-2.213)	<0.001*	259 (49.8)	1.799 (1.438-2.251)	<0.001*
Children	No	632 (73.1)	Reference		307 (35.5)	Reference	
	Yes	393 (83.8)	1.898 (1.423-2.532)	<0.001*	241 (51.4)	1.918 (1.526-2.410)	<0.001*
Education level	Primary/intermediate school	43 (95.6)	Reference		19 (42.2)	Reference	
	High school	259 (74.9)	0.138 (0.033-0.583)	0.070	122 (35.3)	0.745 (0.396-1.401)	0.361
	University and above	714 (76.7)	0.153 (0.037-0.637)	0.010*	399 (42.9)	1.026 (0.560-1.881)	0.933
	Illiterate	9 (81.8)	0.209 (0.026-1.688)	0.142	8 (72.7)	3.649 (0.854-15.600)	0.081
Employment	Student	345 (70.6)	Reference		156 (31.9)	Reference	
	Public sector employee	216 (87.4)	2.908 (1.904-4.443)	<0.001*	135 (54.7)	2.573 (1.879-3.524)	<0.001*
	Privet sector employee	174 (74.4)	1.210 (0.851-1.721)	0.288	105 (44.9)	1.737 (1.261-2.393)	0.001*
	Retired	52 (86.7)	2.713 (1.257-5.856)	0.011*	41 (68.3)	4.606 (2.589-8.196)	<0.001*
	Housewife	97 (77.0)	1.396 (0.883-2.207)	0.153	50 (39.7)	1.404 (0.937-2.104)	0.100
	Unemployed	120 (82.8)	2.003 (1.249-3.214)	0.004*	48 (33.1)	1.056 (0.712-1.568)	0.786
	Self-employed	21 (65.6)	0.797 (0.375-1.695)	0.555	13 (40.6)	1.461 (0.703-3.033	0.310

Public awareness of Wasfati was affected by gender (OR of male participants, 1.8; 95% CI, 1.4-2.2; p<0.001), age >45 (OR, 4.0; 95% CI, 2.8-5.7, p<0.001), Saudi nationality (OR, 1.7; 95% CI, 1.2-2.3; p<0.001), being married (OR, 1.8; 95% CI, 1.4-2.3; p<0.001), and being retired (OR, 4.6; 95% CI, 2.6-8.2; p <0.001) (Table 2). 

Factors affecting awareness with Saha were Saudi nationality (OR, 3.5; 2.6-4.7; p<0.001), working in the public sector (OR, 1.8; 95% CI, 1.2-2.6; p=0.004), being retired (OR, 2.2; 95% CI, 1.0-4.5, p=0.040), and being unemployed (OR, 1.7; 95% CI, 1.1-2.8; p=0.020). Table 3 shows the factors affecting awareness with Moed application, including age >45 years (OR, 2.3; 1.5-3.6; p<0.001), Saudi nationality, being married with children, working in the public sector, and being retired or unemployed (Table 3).

**Table 3:** Factors influencing awareness of the Ministry of Health e-health applications Seha and Moed * indicates a significance based on a significance level of <0.050

N=1333		Seha			Moed		
		Yes (%)	Odds ratio (95% CI)	p-value	Yes (%)	Odds ratio (95% CI)	p-value
Gender	Female	718 (77.1)	Reference		663 (71.2)	Reference	
	Male	290 (72.1)	0.768 (0.589-1.003)	0.052	306 (76.1)	1.288 (0.984-1.687)	0.065
Age	18-24	428 (72.7)	Reference		389 (66.0)	Reference	
	25-35	299 (77.7)	1.308 (0.968-1.766)	0.080	301 (78.2)	1.842 (1.370-2.477)	<0.001*
	36-45	152 (79.2)	1.429 (0.965-2.117)	0.074	142 (74.0)	1.460 (1.014-2.103)	0.042*
	>45	129 (77.2)	1.277 (0.852-1.914)	0.236	137 (82.0)	2.348 (1.527-3.610)	<0.001*
Nationality	Non-Saudi	124 (53.7)	Reference		111 (48.1)	Reference	
	Saudi	884 (80.2)	3.499 (2.597-4.714)	<0.001*	858 (77.9)	3.802 (2.831-5.104)	<0.001*
Marital status	Single	615 (75.6)	Reference		558 (68.6)	Reference	
	Married	393 (75.6)	0.996 (0.771-1.287)	0.977	411 (79.0)	1.723 (1.331-2.230)	<0.001*
Children	No	645 (74.7)	Reference		591 (68.4)	Reference	
	Yes	363 (77.4)	1.163 (0.892-1.516)	0.265	378 (80.6)	1.919 (1.465-2.514)	<0.001*
Education level	Primary/intermediate school	36 (80.0)	Reference		33 (73.3)	Reference	
	High school	253 (73.1)	0.680 (0.315-1.466)	0.325	243 (70.2)	0.858 (0.426-1.727)	0.668
	University and above	710 (76.3)	0.803 (0.381-1.693)	0.565	683 (73.4)	1.001 (0.509-1.970)	0.997
	Illiterate	9 (81.8)	1.125 (0.206-6.142)	0.892	10 (90.9)	3.636 (0.420-31.506)	0.241
Employment	Student	354 (72.4)	Reference		316 (64.6)	Reference	
	Public sector employee	203 (82.2)	1.759 (1.201-2.577)	0.004*	204 (82.6)	2.597 (1.781-3.789)	<0.001*
	Privet sector employee	171 (73.1)	1.035 (0.729-1.469)	0.847	159 (67.9)	1.161 (0.833-1.616)	0.378
	Retired	51 (85.0)	2.161 (1.035-4.511)	0.040*	58 (96.7)	15.877 (3.831-65.797)	<0.001*
	Housewife	87 (69.0)	0.851 (0.555-1.303)	0.458	90 (71.4)	1.369 (0.892-2.101)	0.151
	Unemployed	119 (82.1)	1.745 (1.093-2.788)	0.020*	119 (82.1)	2.506 (1.577-3.981)	<0.001*
	Self-employed	23 (71.9)	0.975 (0.440-2.160)	0.949	23 (71.9)	1.399 (0.633-3.091)	0.406

## Discussion

This study highlighted multiple factors that affect public awareness and satisfaction with multiple e-health applications used in Saudi Arabia. It was apparent that age, gender, education, and nationality played key roles in the awareness of e-health applications. This study investigated multiple services; however, changes occurred as the application Moed (or Mawed) merged with the Sehhati platform, and the relatively new application, Tawaklna, was developed during the pandemic [15].​ The purpose of this study was to assess public awareness and satisfaction and to design specific strategies to increase awareness and usability of such important services. 

With the goals of Saudi vision 2030, one of the major approaches for improving the delivery of healthcare in Saudi Arabia is the adoption of digital health. As the provision of healthcare through traditional methods has some limitations in terms of access and quality of care, the introduction of technology and the use of e-health services could be ways to overcome these deficiencies. Patient satisfaction with some MOH services has been evaluated previously. Previous studies have reported inconsistent findings on the satisfaction of the Saudi population, as some studies reported high satisfaction ​[11] and utilization​ [15], and others reported reluctance. 

While Saudi Arabia has taken a positive direction regarding the enactment of e-health, it must undertake additional measures to improve its implementation. Future plans for the development of e-health states in the Kingdom should be approached holistically, taking into consideration the different parties affecting the process of successful implementation. Regulatory bodies' responsibility, healthcare providers' roles, and patients' acceptance and literacy must be emphasized. Appropriate information and communications technology infrastructure readiness, financing, investment, legalizations, and policies encouraging the cultural adaptation of e-health are highly impactful and crucial for development. Concerns regarding confidentiality and privacy are among the pressing factors that affect users' acceptance and adaptations to e-health systems. Therefore, the incorporation of governmental regulatory bodies would be crucial in promoting confidence in the e-health system. Adapting the delivery of e-health services to Saudi cultural and social values will bridge essential trust in using big data and further impact the development and adoption of e-health systems [16].

This study is the first of its kind in Saudi Arabia, combining multiple MOH e-health services evaluations. It provides insights into the readiness of the population to embrace the transition to e-health. It also has a wide range of population characteristics, which may enhance the generalizability of this study. The limitations of this study include its cross-sectional nature and lack of information on the diseases of the participants, which could have been beneficial to know. 

## Conclusions

This study indicates the readiness of the population for transition to e-health; however, multiple groups of the population need more awareness and education regarding the services provided. This study could serve as a baseline for future educational campaigns arranged by the MOH to transition to e-health under the Saudi 2030 vision. 
